# Serological Survey and Factors Associated with* Toxoplasma gondii* Infection in Domestic Goats in Myanmar

**DOI:** 10.1155/2016/4794318

**Published:** 2016-01-21

**Authors:** Saw Bawm, Wint Yi Maung, Myat Yee Win, May June Thu, Hla Myet Chel, Tin Aye Khaing, Soe Soe Wai, Lat Lat Htun, Tin Tin Myaing, Saruda Tiwananthagorn, Makoto Igarashi, Ken Katakura

**Affiliations:** ^1^Department of Pharmacology and Parasitology, University of Veterinary Science, Nay Pyi Taw 15013, Myanmar; ^2^Livestock Breeding and Veterinary Department (Head Quarter), Nay Pyi Taw 15011, Myanmar; ^3^Unit of Risk Analysis and Management, Research Centre for Zoonosis Control, Hokkaido University, Sapporo 060-0818, Japan; ^4^Nay Pyi Taw Development Committee, Nay Pyi Taw 15011, Myanmar; ^5^Myanmar Veterinary Association, Yangon 11011, Myanmar; ^6^Department of Veterinary Biosciences and Veterinary Public Health, Faculty of Veterinary Medicine, Chiang Mai University, Chiang Mai 50100, Thailand; ^7^National Research Center for Protozoan Diseases, Obihiro University of Agriculture and Veterinary Medicine, Obihiro, Hokkaido 080-8555, Japan; ^8^Laboratory of Parasitology, Graduate School of Veterinary Medicine, Hokkaido University, Sapporo 060-0818, Japan

## Abstract

Goat farming is important for the livelihood of millions of rural people because it contributes to food security and creation of assets. However, infection of goats with* Toxoplasma gondii* could be a source of parasite transmission to humans. The information on* T. gondii* infection of goat was not reported yet in Myanmar. A total of 119 goat serum samples were collected from three cities in the central region of Myanmar for* T. gondii* antibody survey. With the occurrence value obtained in this first study, a second one, more complete, with larger number (162) of animals and properties, was carried out and the risk factors and prevalence were determined. In both studies the samples were analyzed by the LAT. Of these, 32 (11.4%) samples were showed to be positive. The infection was associated with the presence of cats at the farm (odds ratio [OR] = 4.66, 95% confidential interval [CI] = 1.03–21.06), farming with different animal species (sheep, cattle, and pigs) (OR = 4.33, 95% CI = 1.57–11.94), and farming without good management practices (OR = 0.23, 95% CI = 0.06–0.83). This is the first* T. gondii* prevalence study in goats in the country.

## 1. Introduction

*Toxoplasma gondii* is a zoonotic protozoan parasite, and the meat of infected food animals (pigs, goats, sheep, or poultry) is one of the most important potential sources of human toxoplasmosis [1]. In humans, transplacental transmission of the parasite in pregnant women often leads to death or damage to the central nervous system of the fetus [2]. In Myanmar, serological studies showed that the prevalence of* T. gondii* infection in humans was 31.8–41.0% [3–5], but infection sources are poorly understood.

Infection of domestic animals, including cattle, pigs, sheep, goats, and chickens, with* T. gondii* occurs widely around the world, resulting in insignificant reproductive and economic losses [2]. Ingestion of undercooked meat and unpasteurized milk and cheese from infected goats are potential sources of infection in humans [6–8]. In the central region of Myanmar, farming of small ruminants, especially goats (*Capra hircus*), is of economic importance. However, the prevalence of* T. gondii* infection among domestic animals is unknown in Myanmar. This study aimed to determine the seroprevalence of* T. gondii* antibodies in domestic goats in the central region of Myanmar and assess risk factors for infection.

## 2. Materials and Methods

### 2.1. Study Areas and Study Animals

In an initial study, blood samples were collected from 119 goats on eight farms near three cities, Nay Pyi Taw (located within 19°75′N and 96°13′E), Mandalay (located within 21°97′N and 96°08′E), and Pyin Oo Lwin (located within 22°03′N and 96°47′E), of Myanmar in January 2009. For a cross-sectional study in June 2013, blood samples were collected from 162 goats on 15 farms in Pyawbwe Township (located within 20°35′0′′N and 96°4′0′′E). The sample size in Pyawbwe was determined by taking into account the assumed prevalence of anti-*T. gondii* antibodies (20.7%), which was estimated from the ratio of positive samples in the initial study with an exact binomial confidence interval of 95% [9]. The total goat population in the municipality of Pyawbwe was 24,500 goats according to Pyawbwe Township Livestock Breeding and Veterinary Department in the year of 2012.

In these study areas, average herd size is 10–30 goats per farm and they were raised under semi-intensive system. Stratified systematic sampling method was used for sample collection. A questionnaire was used to record the general characteristics of the goats including age, sex, and breed (Kalahari or Jamnapari). In addition, environmental data were recorded, including the presence of cats on the farm, farming with other animals (sheep, cattle, pigs, and chickens), and farming with good management practices (good hygiene, proper feeding, regular vaccination, and deworming).

This project was approved by the Ministry of Livestock, Fisheries and Rural Development and University of Veterinary Science, Myanmar. Informed consent was obtained from all owners of the goats.

### 2.2. Blood Sampling and Serological Examination

Blood samples were collected from the jugular vein of goats and dropped on blood sampling paper (Nobuto Strip, ADVANTEC^*®*^, Tokyo, Japan). The filter paper strips were air-dried and kept in 4°C with a desiccant until use. Serum components were eluted from the strip by soaking the blood-absorbed area in 400 *μ*L of 0.01 M phosphate buffered saline (PBS), pH 7.4, for 60 min at room temperature, in accordance with the manufacturer's instructions, to make a solution equivalent to a 10-fold dilution of the serum. The eluent was further diluted with PBS and tested using a Latex Agglutination Test (LAT) kit (Toxocheck-MT; Eiken Chemical, Tokyo, Japan). The LAT was carried out in a 96-well microplate with twofold dilutions from 1 : 16 to 1 : 4,096. The plate was shaken for 2 min and then incubated at room temperature overnight. The test was considered positive reaction when a layer of agglutinated latex beads was formed in a well at dilutions of 1 : 64 or higher [10].

### 2.3. Statistical Analysis

Statistical analysis was performed using Epi Info software version 7.1.2 (Centers for Disease Control and Prevention: http://wwwn.cdc.gov/epiinfo/7/). Yates' corrected Chi-square test was used for comparison of the frequencies among groups. The difference in prevalence among groups was analyzed statistically by Chi-squared independence. Risk was expressed as an odds ratio (OR) with 95% confidence interval (CI). Statistical significance was set at a* p* value of <0.05.

## 3. Results and Discussion

The initial survey (2009) in Mandalay, Nay Pyi Taw, and Pyin Oo Lwin showed occurrence of* T. gondii* infection that ranged from 4.7% to 20.7% (Table 1). In the second study (2013) in Pyawbwe, the seroprevalence was 11.8%. In total, anti-*T. gondii* antibodies were detected in 32 of 281 (11.4%) goats (Table 1), and reciprocal titers ranged from 64 to 2,048, although no obvious peak of titers was observed (Table 2).

Risk factors associated with seropositivity against* T. gondii* infection in Pyawbwe are shown in Table 3. Statistical analysis using Chi-square test revealed that the presence of anti-*T. gondii* antibodies in goats was associated with the presence of cats on the farm (*p* = 0.038, OR = 4.66, 95%, and CI = 1.03–21.06), farming with different animal species (such as sheep, cattle, and pigs) (*p* = 0.003, OR = 4.33, and 95% CI = 1.57–11.94), and management without good practices (poor hygiene, lack of proper feeding, regular vaccination, and deworming) (OR = 0.23; 95% CI = 0.06–0.83). Age, sex, and breed of the goats presented no association with infection (Table 3).

A number of recent epidemiological studies showed the worldwide spread of* T. gondii* in goats. In Myanmar, the present study indicated that the seroprevalence of* T. gondii* in goats was 11.4%. In the neighboring countries, it was 27.9% in Thailand [11], 61.0% in Bangladesh [12], 41.3% in India [13], 25.4% in Pakistan [14], and 3.8–30.8% in China [15, 16]. The difference in the seroprevalence rates in these Asian countries could be due to differences in diagnostic techniques, sample size, age of the animals, and climatic and management variations from one region to another.

The risk factors for* T. gondii* infection in goats have been analyzed in different countries. In Brazil, risk factors were detected as age over 36 months, use of a pen, pure breed animals, the presence of cats alongside the herd, intensive or semi-intensive management systems, and a lack of mineral supplementation [17, 18]. In Greece, risk factors comprised intensive or semi-intensive management, feeding concentrate, and providing water from the public supply [19]. Furthermore, access of stray cats to animals' water and the presence of wild felids were potential risk factors for seroprevalence of* T. gondii* in dairy sheep flocks in Italy [20] and Ethiopia [21], respectively. Taken together, the presence of cats, a large flock size, and the method of disposing of aborted fetuses are potential risk factors for* T. gondii* infection in goat and sheep. The present study also showed that one of the risk factors was the presence of cats on the farm, and this result was in agreement with those from previous studies.

Cats act as the final host in the life cycle of* T. gondii* and excrete oocysts in their feces that can be a source of infection for humans and other animals [2]. Oocysts eliminated by cats remain infective for months to years in moist, shaded, and temperature-regulated environmental conditions [22]. In this study almost all farms had different animal species (sheep, cows, oxen, pigs, and poultry) living in the same environment and via the movement of those animals the infection and transmission could be facilitated. The feces and urine of animals, waste food, and water supplied to animals might contribute to the maintenance of viability of oocysts. In addition, farming without good management practices was also identified as a risk factor in this study. Poor hygiene conditions and animals management implicate an increased risk for* T. gondii* infection. Studies on the seroprevalence of* T. gondii* infection in humans and other domestic animals are necessary to better understand the epidemiology of* T. gondii* in Myanmar.

Livestock are a key asset for farmers in Myanmar, and more than half of the national sheep and goat population were found in the central region, the studied area. In order to fight poverty in Myanmar it is important to develop goat related industries and* T. gondii*, an important zoonotic pathogen, needs to be controlled once this coccidian could be present in the meat, milk, and cheese of infected goats [6–8].

## 4. Conclusions

This is the first report on the seroprevalence of* T. gondii* infection in domestic goats in Myanmar that found a prevalence value of 11.4% and presence of cats on the farms, farming with different animal species, and farming without good management practices presented association with the infection.

## Figures and Tables

**Table 1:** Seroprevalence of *T. gondii *infection in goats in four cities in Myanmar.

City	Sampling time	Number of samples (number of farms)	Number of positive
Mandalay	Jan 2009	50 (2)	5 (10.0%)
Nay Pyi Taw	Jan 2009	40 (3)	3 (4.7%)
Pyin Oo Lwin	Jan 2009	29 (3)	6 (20.7%)
Pyawbwe	June 2013	162 (15)	18 (11.8%)

Total		281 (23)	32 (11.4%)

**Table 2:** *Toxoplasma gondii *antibody titers, by latex agglutination test (cut-off ≥ 64) in 281 goat sera in Myanmar.

Titer	Number of positive	% of positive
<1 : 32	215	76.5
1 : 32	34	12.1
1 : 64	8	2.9
1 : 128	9	3.2
1 : 256	3	1.1
1 : 512	3	1.1
1 : 1,024	6	2.1
1 : 2,048	3	1.1

(There were 7 farms out of 15 farms that were showed to be positive with at least one animal.)

**Table 3:** Factors associated with *T. gondii *infection in goats in Pyawbwe City.

Category	Sample size	Number of positive (%)	Odds ratio (95% CI)	*p*
Age of animal (years)				
0-1	9	1 (11.1)	—	
1-2	102	10 (9.8)	1.15 (0.13–10.16)	
>2	51	7 (13.7)	0.68 (0.24–1.91)	0.550
Sex of animal				
Male	56	5 (8.9)	—	
Female	106	13 (12.3)	0.70 (0.24–2.08)	0.522
Breed				
Jade ni (Kalahari)	7	2 (28.6)	—	
Htein san (Jamnapari)	155	16 (10.3)	3.47 (0.62–19.40)	0.134
Presence of cats				
Yes	107	16 (15.0)	4.66 (1.03–21.06)	0.038
No	55	2 (3.6)	—	
Farming with different species (sheep, cattle, pigs, and poultry)				
Yes	36	9 (25.0)	4.33 (1.57–11.94)	0.003
No	126	9 (7.1)	—	
Good management practices				
Yes	70	3 (4.3)	—	
No	92	15 (16.3)	0.23 (0.06–0.83)	0.016
